# Physiology and Transcriptional Analysis of ppGpp-Related Regulatory Effects in Streptomyces diastatochromogenes 1628

**DOI:** 10.1128/spectrum.01200-22

**Published:** 2022-12-08

**Authors:** Yang Song, Xiangli Zhang, Zixuan Zhang, Xuping Shentu, Xiaoping Yu

**Affiliations:** a Zhejiang Provincial Key Laboratory of Biometrology and Inspection and Quarantine, College of Life Science, China Jiliang University, Hangzhou, China; The University of Tennessee Knoxville

**Keywords:** *Streptomyces*, ppGpp, RelA/SpoT, stringent response, primary metabolism, secondary metabolism

## Abstract

ppGpp is a ubiquitous small nucleotide messenger that mediates cellular self-protective responses under environmental stress. However, the mechanisms of ppGpp that control transcription and other metabolic processes depend on the species, and ppGpp regulates the same process via different mechanisms. The level of ppGpp is regulated by RelA/SpoT homolog (RSH) enzymes that synthesize and hydrolyze the alarmone. Here, we constructed a ppGpp^0^ strain and monitored the effects of ppGpp on the transcriptional level, physiology, and secondary metabiotic production in the antibiotic producer Streptomyces diastatochromogenes 1628. The results showed the cell division and growth of ppGpp^0^ increased by measurement of gene transcription and DCWs. The utilization of nitrogen was affected depending on the nitrogen type with a significantly higher DCW of the ppGpp^0^ mutant in the medium supplied with the yeast extract and a lower growth rate in the inorganic nitrogen ammonium salt. The ppGpp-mediated stringent response could not affect the usage of carbon resources. More importantly, ppGpp^0^ inhibited the expression of antibiotic clusters and the production of toyocamycin and tetramycin P. The antibiotic resistance was also significantly downregulated in the ppGpp^0^ mutant. In conclusion, this study showed detailed changes in ppGpp-mediated stringent responses on S. diastatochromogenes 1628 cell growth, nutrient utilization, morphological characteristics, antibiotic production, and resistance, which will provide insights into the role of ppGpp in *Streptomyces*.

**IMPORTANCE** The ppGpp-mediated stringent response is widely distributed in Escherichia coli, Bacillus subtilis, *Streptomyces*, Staphylococcus aureus, etc. Stringent responses give strains the ability to resist environmental stresses, and survival from nutrition starvation, virulence, long-term persistence, biofilm formation, and gut colonization. ppGpp has many targets in cells and can reprogram DNA replication, transcription, ribosome biogenesis and function, and lipid metabolism. However, the mechanism of ppGpp to control transcription and other metabolic processes depends on the bacterial species and regulates the same process via a different mechanism. In *Streptomyces*, how ppGpp regulates the transcription remains to be elucidated. However, because ppGpp regulates many genes involved in primary and secondary metabolism, we compared the transcription and cell division, cell growth, morphological differentiation, antibiotic resistance, and secondary synthesis in the wild-type S. diastatochromogenes and ppGpp^0^ strains.

## INTRODUCTION

The Streptomyces diastatochromogenes 1628 could produce a wide variety of secondary metabolites, including toyocamycin and tetraene macrolides. Toyocamycin is a nucleotide antibiotic with potential activity against several phytopathogenic fungi and has been recognized as a valuable fungicide in the agricultural industry (1). The tetraene macrolides, such as tetramycin A, tetramycin P, and tetrin B, exhibit biological activities, including antifungal, anticancer, and immunosuppressant activities, and can be used in medicine, veterinary medicine, agriculture, and food preservation (2). Thus, S. diastatochromogenes 1628 is a promising antibiotic producer. Commonly, these secondary metabolic pathways were silent in the nutrition enough condition, and active during the nutrition shift to improve the ecological and competitive ability.

The secondary metabolism in the *Streptomyces* is subject to the regulation of many global or pathway-specific regulators, such as the signal molecules or factors. Among them, the small nucleotide messenger alarmone guanosine tetraphosphate (ppGpp), which induces the stringent response of bacteria, is a ubiquitous stress signaling pathway that enables the cell to survive numerous environmental stresses during its life cycle. The ppGpp has many targets, including transcription, ribosome biogenesis, purine metabolism, and morphological differentiation (3). The level of ppGpp *in vivo* is regulated by the proteins of the RelA and SpoT homolog (RSH) family. Rel is a bifunctional enzyme, which is capable of both synthesizing (catalyzed by the synthetase domain, SYNTH) and hydrolyzing (catalyzed by the hydrolase domain, HD) the (p)ppGpp alarmone. In addition, there are single-domain RSHs coded by the bacteria called small alarmone synthetases (SAS) and small alarmone hydrolases (SAH) (4). In S. diastatochromogenes DSM 40608, four enzymes control the level of ppGpp together, the RshA [HS] and Rel [HS], which are long RSHs with bifunctional domain for synthesis and hydrolysis, the small alarmone synthetase ActRel2 [S], and the small alarmone hydrolase PbcSpo [H] (5–7). However, *Streptomyces* has a high frequency of mutants in the genome, and there is considerable genome diversity within the naturally occurring populations of strains collected from different geographic locations. Thus, the RSH family genes in S. diastatochromogenes 1628 should be analyzed before controlling the ppGpp level.

The ppGpp can activate or inhibit bacterial metabolism and physiology during the stringent response, and some of the ppGpp-binding targets are common, while others are specific to the lifestyle and niche of species (8, 9). For example, the ppGpp is recognized to activate the amino acid metabolism and secondary metabolism. In wild-type Streptomyces coelicolor, the production of polyketide antibiotic actinorhodin increased by 180-fold due to the accumulation of ppGpp (10). Conversely, the ppGpp could negatively regulate secondary metabolism to decrease the production of clavulanic acid and cephamycin C in the S. clavuligerus (11). Therefore, the regulatory effect of ppGpp on antibiotic production in the S. diastatochromogenes 1628 remains to be revealed.

Because of the large difference in the genome that existed in the species of *Streptomyces*, the genome of S. diastatochromogenes 1628 was sequenced (unpublished data) and genomic analysis suggested that S. diastatochromogenes 1628 had low homology with the S. coelicolor and high homology with S. albulus. The secondary metabolic clusters in S. diastatochromogenes 1628 were similar to that in the S. hygrospinosus variant beijingensis. The toy cluster was identified, and 13 genes were contained and regulated by the pathway-specific ToyA (1) and global regulator AdpA (10). The tetraene macrolide pathway was arranged as the same gene organization but different in nucleotide and amino acid sequences with the gene cluster of polyene macrolide antibiotic tetramycin in the S. hygrospinosus variant beijingensis (2). However, there has been little research on the effect of ppGpp on the secondary metabolism in the S. diastatochromogenes 1628 or the status of this strain in producing potential antibiotics.

Further, it was also necessary to compare the regulation of ppGpp on the cell division, growth, transcription, translation, and morphological differentiation to the ppGpp-deficient strains and wild-type strains because the development of *Streptomyces* was related to the production of antibiotics. Therefore, in this study, the influence of ppGpp-mediated stringent response on the growth, antibiotic production, or tolerance of S. diastatochromogenes 1628 was studied systemically, which will bring insight into the function of stringent response in the *Streptomyces*.

## RESULTS

### Effect of ppGpp on cell division and growth.

**FIG 1:**
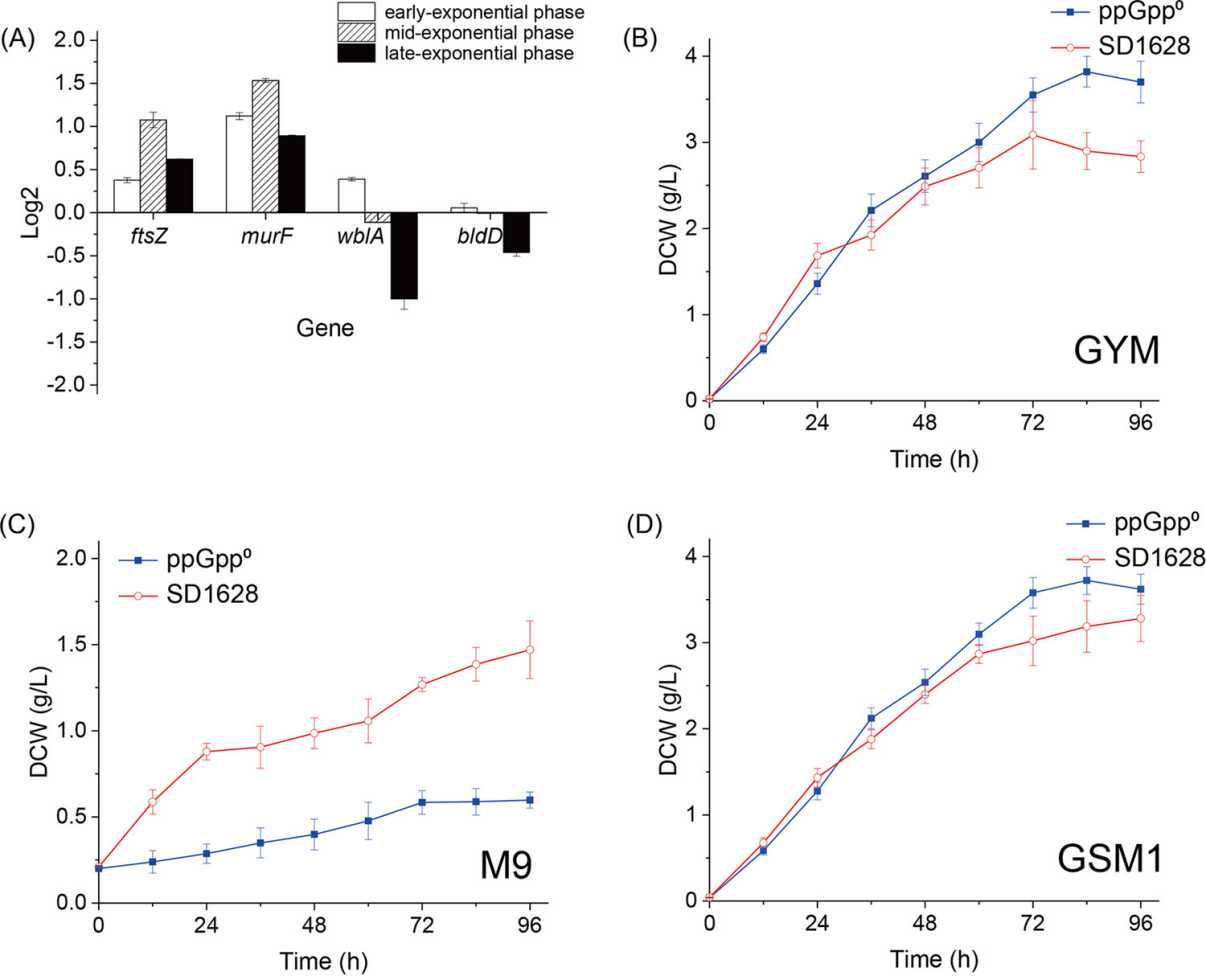
The effect of interrupting ppGpp on the growth and transcription level in S. diastatochromogenes 1628. The transcription level of genes regulating cell division and cell differentiation were compared (A). The bacterial growth was measured by the dry cell weight of S. diastatochromogenes 1628 (SD1628), which were inoculated at 28°C in three mediums: GYM (B), M9 (C), and GSM1 (D).

Whole-genome sequencing analysis of S. diastatochromogenes 1628 revealed that there were three enzymes to control ppGpp level, PbcSpc [H], RshA [HS], and Rel [HS]. The *ActRel2* [S] gene could not be detected in the genome of S. diastatochromogenes 1628, and no PCR product was detected by using the primer designed according to the *ActRel2* [S] sequences from S. diastatochromogenes DSM 40608 (5). The proteins encoded by genes *Rel* [HS] and *RshA* [HS] control the synthesis of ppGpp *in vivo* and each had only one copy in the genome. Srivatsan et al. (6) found that the deletion of one gene could lead to secondary suppressor mutations of other RSH genes to avoid the overaccumulation of ppGpp. Therefore, we first deleted the *Rel* gene in S. diastatochromogenes 1628. Then, by screening the mutants, one clone has a mutant sequence of the *RshA* gene (a frameshift Leu295), which meant that interruption of *Rel* [HS] and *RshA* [HS] genes resulted in a strain (ppGpp^0^) without the ability to generate ppGpp (Fig. S1 in Supplemental File 1). However, the *PbcSpc* [H] was not mutated, indicating the strain could still hydrolyze ppGpp. Because the influence of ppGpp on cellular processes is highly complex and show large differences between bacterial species, the detailed transcriptional changes of related genes and molecular mechanism were necessary to investigate. *ftsZ* (encoding the tubulin homolog) and *murF* (encoding the muscle ring-finger protein) participate in cell division and peptidoglycan biosynthesis, which are affected by the cellular signal molecular ppGpp (12). By comparing the transcriptional level of *ftsZ* and *murF* in the wild-type and mutant strains via quantitative (q)PCR, the mutant strain showed 2-fold higher transcriptional levels at the mid-exponential and late exponential phases, which indicated the negative regulatory influence of RSHs on the cell division (Fig. 1A).

The growth of *Streptomyces* includes cell division, cell differentiation, and morphological conversion during the life span. For growth and reproduction, the *Streptomyces* undergo three morphologically and physiologically distinct forms, and BldD controls the switch from the extension of vegetative hyphae to the erection of aerial hyphae, meaning the conversion from the exponential phase to the stationary phase (13). *WhiA* gene regulates the division of aerial hyphae into chains of prespore compartments (14). Thus, the transcriptional level of *BldD* and *WhiA* brought insight into the developmental transitions of *Streptomyces*. The quantitative (q)PCR results (Fig. 1A) indicated that the transcriptional level of *BldD* and *WhiA* showed no significant difference at the early exponential phase and mid-exponential phase in the wild-type and ppGpp^0^ strains. While the transcriptional level of *BldD* and *WhiA* in ppGpp^0^ decreased significantly by 0.92-fold and 2-fold in the late exponential phase. This phenomenon suggested that the interruption of ppGpp could extend the exponential phase and produce more vegetative hyphae for the ppGpp^0^ mutant than the wild-type strain.

The effect of RSHs on cell division and differentiation should contribute to the change of bacterial growth, thus the DCWs of S. diastatochromogenes 1628 were monitored in the three types of mediums, including glucose yeast extract medium (GYM), M9, and Gauze's Synthetic Medium No. 1 (GSM1). The growth of ppGpp^0^ increased compared with that of the wild-type strains in the GYM and GSM1 medium. In the GYM medium, the DCW of the wild-type was higher than the ppGpp^0^ mutant at the first 24 h, but the growth of ppGpp^0^ became significantly superior to the wild-type after 36 h. The highest DCW of ppGpp^0^ reached 3.82 g/L at 84 h (Fig. 1B). The similar growth trend was also observed in the GSM1 medium, and the highest DCW was 3.72 g/L and 3.28 g/L for the ppGpp^0^ and wild-type strains, respectively (Fig. 1D). However, in the M9 medium, a limited nutrition condition, the growth of the wild-type strain was much higher than the ppGpp^0^ mutant, and the DCW at 96 h was significantly lower than that in the GYM and GSM1 medium. The highest DCW was only 1.47 g/L and 0.59 g/L for the wild-type and ppGpp^0^ strains, respectively (Fig. 1C). The results indicated that the interruption of ppGpp synthesis negatively affected the cell growth of S. diastatochromogenes 1628 in the nutrition-limited medium. Because ppGpp was the signal molecular of the stringent response, the defect of stringent response was related to the usage of nutrition in the medium in *Streptomyces*.

**FIG 2:**
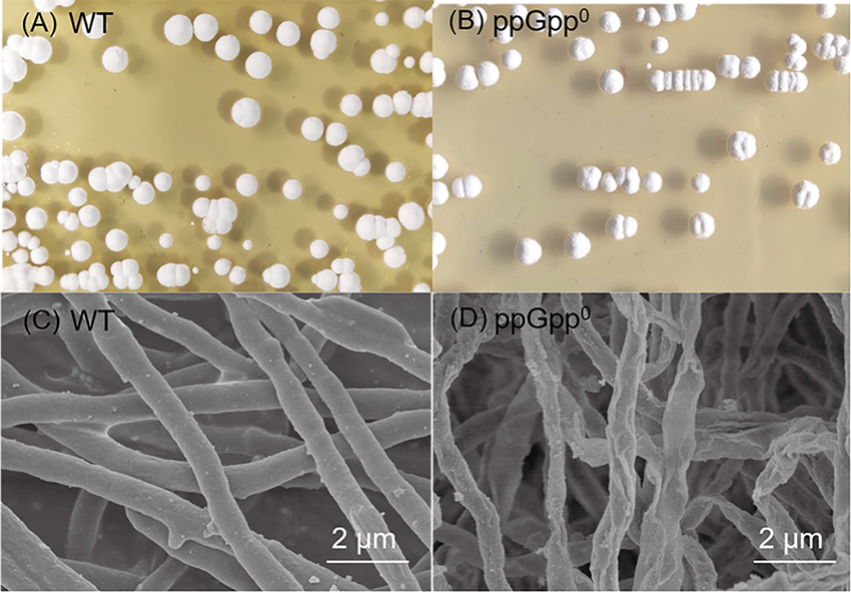
The difference of morphological differentiation on the GYM agar plates and GYM liquid medium after cultivation for 4 days at 28°C. WT, wild-type S. diastatochromogenes 1628. ppGpp^0^, the wild-type strain carrying the interruption of RSH genes.

The morphological characteristics of the wild-type and ppGpp^0^ strains on GYM agar plates (Fig. 2A and B) or in the GYM liquid medium (Fig. 2C and D) were also investigated. The aerial mycelium appeared white to gray and colorless on the reverse side of the plate. There were pits in the middle of the ppGpp^0^ colonies, but the colonies of wild-type strain were rounded and smoothed on the surface. The scanning electron microscopy (SEM) results showed that the aerial mycelium of ppGpp^0^ was wrinkled, which was related to the interruption of ppGpp production.

### Effect of stringent response on the carbon and nitrogen source usage.

**FIG 3:**
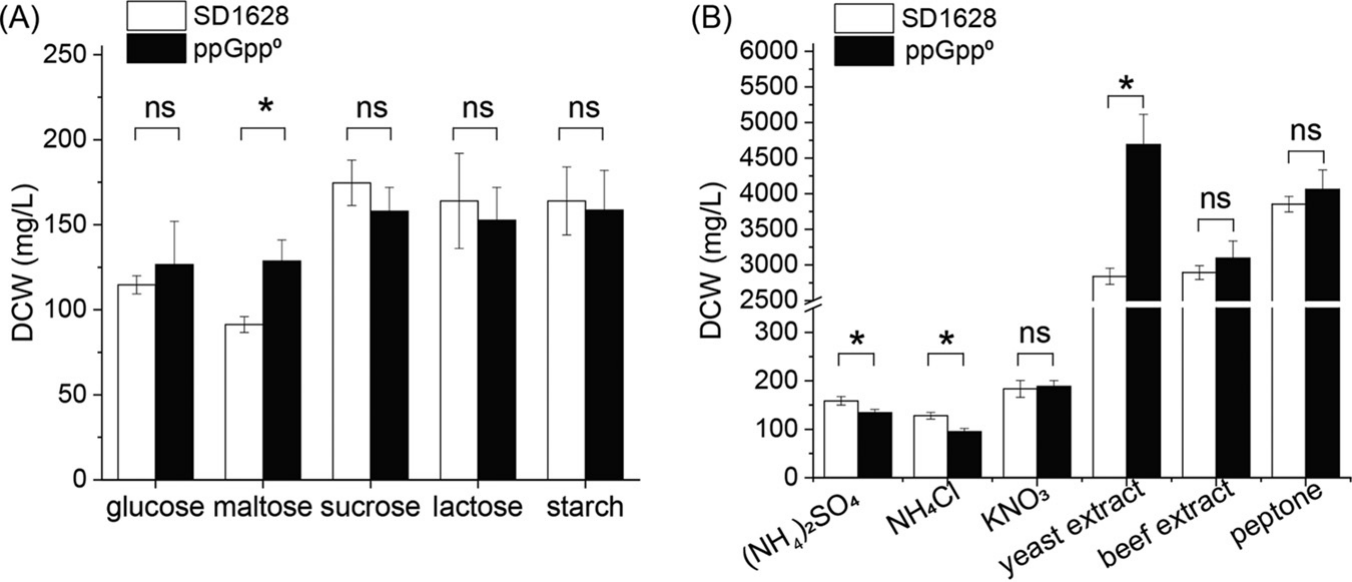
The regulatory effect of ppGpp on the carbon and nitrogen resources. The DCWs of wild-type (SD1628) and ppGpp^0^ were monitored at 28°C in the basic medium supplied with different carbon resources (A) and nitrogen resources (B). The significant difference among the groups was calculated in SPSS, version 25.0 (IBM, Armonk, New York, USA) using the nonparametric *t* test, and *P* < 0.05 was considered a significant difference.

Based on the regulatory effect of ppGpp on cell growth, it was predicted that the ppGpp^0^ mutant might affect the usage of carbon and nitrogen sources in the medium. Therefore, the growth of S. diastatochromogenes 1628 in the mediums with glucose, maltose, sucrose, lactose, and starch as the sole carbon source was measured, and the DCWs of S. diastatochromogenes 1628 in the organic and inorganic nitrogen sources medium were compared. For the carbon usage, the DCW of ppGpp^0^ was significantly higher than that of the wild-type strain in the maltose (*P* < 0.05), while the DCWs at 96 h did not show a difference between the S. diastatochromogenes 1628 and ppGpp^0^ strains in other carbon mediums. This indicated that the ppGpp-mediated stringent response could not affect the usage of carbon resources (Fig. 3A).

Notably, the growth of *Streptomyces* was affected by the organic nitrogen and inorganic nitrogen resources. S. diastatochromogenes 1628 and ppGpp^0^ strains were grown at 28°C in a basic medium with inorganic and organic nitrogen, and the DCWs in the organic nitrogen were much higher than that in the inorganic nitrogen. The DCWs in the inorganic medium were 95.33 to 188.67 mg/L, and the growth of S. diastatochromogenes 1628 was significantly higher than the ppGpp^0^ mutant in the basic medium supplied with (NH_4_)_2_SO_4_ and NH_4_Cl, which meant that the ppGpp could increase the ability to use inorganic nitrogen of ammonium (Fig. 3B). While the DCWs of these two strains were about 2.84 to 4.68 g/L at 96 h in the organic nitrogen resources (yeast extract, beef extract, and peptone; 20 g/L), the DCWs of ppGpp^0^ was significantly higher than the wild-type strain in the yeast extract (*P* < 0.05) and reached 4.688 g/L. However, there was no significant difference in the basic medium supplied with beef extract and peptone, although the average DCWs were relatively higher than ppGpp^0^ (Fig. 3B). The result meant that the cell division of ppGpp^0^ was faster than that of the wild-type strain in the sufficient nitrogen condition.

### ppGpp^0^ exhibited upregulated transcription of translation-related genes.

**FIG 4:**
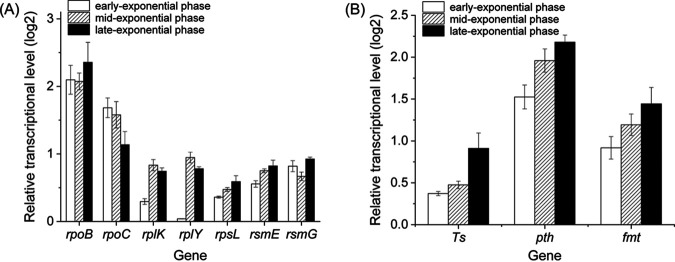
The transcriptional abundance of genes (revolving in the transcription and translation processes) in the ppGpp^0^ mutant compared with the wild-type strain (SD1628).

*rpoB* and *rpoC* encoded the RNA polymerase β and β’ subunit, which is related to the transcription process in the strains. The expression of *rpoB* and *rpoC* increased more than 2-fold in the ppGpp^0^ mutant strain at the early-, mid-, and late- exponential phases compared with the S. diastatochromogenes 1628, which suggested that the synthesis of RNA polymerase was upregulated without the stringent response regulation. The increased synthesis of RNA polymerase meant the transcriptional process increased in the ppGpp^0^ mutant strains (Fig. 4A).

*rplY* and *rplK* encoded the ribosomal proteins L25 and L11, respectively, and *rpsL* encoded the ribosomal protein S12. The transcriptional level of these three genes in the ppGpp^0^ increased at the mid-exponential and late exponential phases compared with the wild-type strain (Fig. 4A), indicating that the ability of ribosome protein synthesis was increased by interruption of stringent response. The *rsmE* and *rsmG* encoded the rRNA small subunit methyltransferase genes E and G (15, 16). In the ppGpp^0^ mutant of S. diastatochromogenes 1628, the transcription of *rsmE* and *rsmG* was higher than that of the wild-type strain in all the growth phases, which indicated that the methylguanosine of 16S rRNA genes (catalyzed by RsmE and RsmG) was enough and that more ribosome was synthesized in the ppGpp^0^ mutant strain (Fig. 4A). A more efficient synthesis of protein was to satisfy the faster cell division rate of *Streptomyces*.

The expression of general translation factors, transcriptional elongation factor gene (*Ts*), an aminoacyl-tRNA hydrolase gene (*pth*), and a methionyl-tRNA formyltransferase gene (*fmt*) increased in the ppGpp^0^ strain (Fig. 4B). In addition, the increase of the transcriptional level of these three genes in ppGpp^0^ became more distinct over time and the expression of *Ts*, *pth*, and *fmt* increased by 1.88-fold, 4.53-fold, and 2.72-fold, respectively, in the late growth phase. These results indicated that ppGpp could affect the translation process.

### Effect of the ppGpp^0^ mutant on the secondary metabolism.

**FIG 5:**
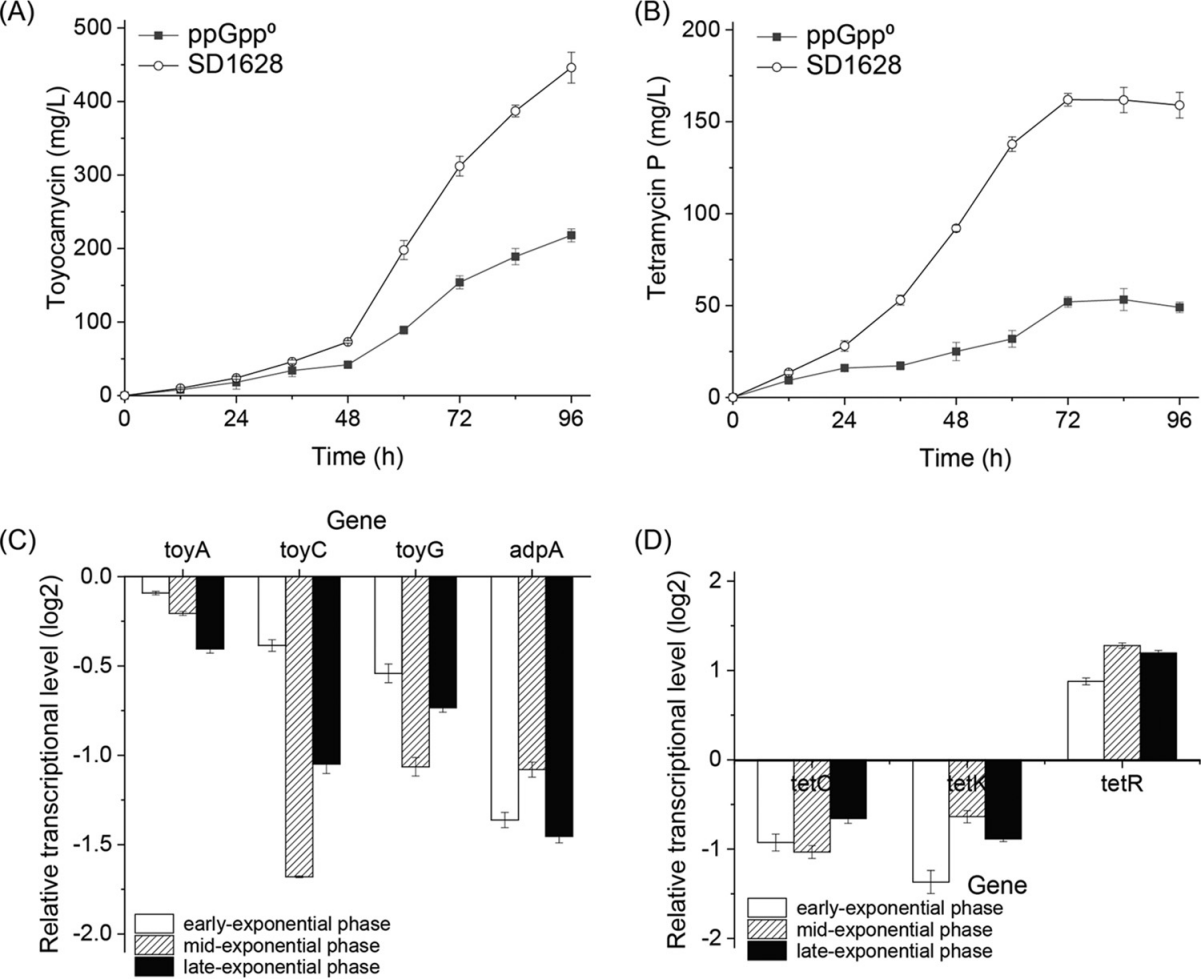
The positive regulatory effect of ppGpp the secondary metabiotic production and gene expression in S. diastatochromogenes 1628. (A and B) The production of toyocamycin and tetramycin P in the GYM medium inoculated at 28°C with 2% of the inoculation. (C and D) The relative fold change in the mRNA levels by qPCR of genes in the toy cluster and tetraene macrolide cluster.

*Streptomyces* produce various types of important bioactive metabolites, and many secondary metabolites were employed clinically as antibiotics. The two categories of secondary metabolites produced by S. diastatochromogenes 1628 have been identified as toyocamycin and tetramycin P, which was synthesized by the toy cluster (toyocamycin) (17) and the tetraene macrolide cluster (tetramycin P) (18). Previous studies showed that the stringent response could positively regulate the synthesis of secondary metabolism. In this study, the relationship between ppGpp-mediated stringent response and the expression of two clusters was tested by monitoring the titer of antibiotics and the transcriptional level of the genes in two clusters (Fig. 5). We found that the titer of toyocamycin in the ppGpp^0^ strain at 84 h (218.42 mg/L) was lower than the wild-type strain at 96 h (446.17 mg/L) (Fig. 5A). At the same time, the production of tetramycin P in ppGpp^0^ (53.24 mg/L) was also lower than the wild-type strain (161.80 mg/L) at 96 h, which indicated that the synthesis of secondary metabiotic was affected by ppGpp (Fig. 5B).

The transcriptional level of structural genes *toyA*, *toyC*, and *toyG* in the toy cluster was downregulated in the ppGpp^0^ mutant at the early, mid-, or late exponential phases, and the gene of toy cluster activator *adpA* (19) reduced 1.47-fold at late exponential phase in the ppGpp^0^ mutant (the same gene in the wild-type strain was used as the reference gene), which indicated the decreased transcription of secondary metabiotic clusters existed in the ppGpp^0^ mutant (Fig. 5C). Similarly, the transcriptional level of genes *tetC* and *tetK* (18) in tetraene macrolide cluster downregulated, while the expression of negatively transcriptional regulator tetR upregulated in the ppGpp^0^ mutant by 2.29-fold at the late exponential phase, which was coherent with lower tetramycin P production (Fig. 5D). Therefore, the ppGpp-mediated stringent positively activated the production of secondary antibiotics, and the ppGpp played a key role in globally activating the transcriptional process of secondary clusters in the S. diastatochromogenes 1628.

### ppGpp^0^ reduced the antibiotic resistance in the S. diastatochromogenes 1628.

**FIG 6:**
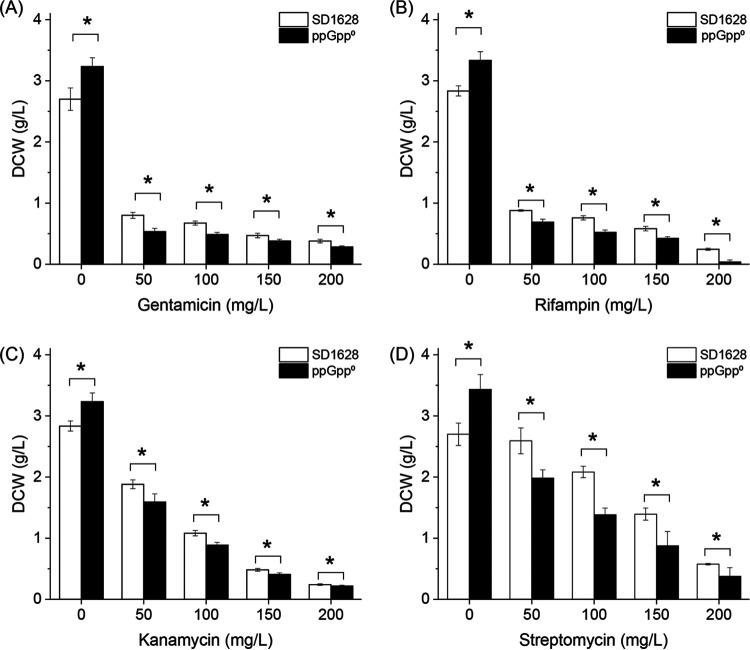
The influence of ppGpp on antibiotic resistance in the wild-type (SD1628) and ppGpp^0^ strains The DCWs were monitored after inoculation for 96 h at 28°C in the GYM medium with increasing concentrations of gentamicin (A), rifampin (B), kanamycin (C), and streptomycin (D).

The stringent response was related to the resistance and adaption of the strains (20). Here, the effect of the ppGpp on the antibiotic resistance level in S. diastatochromogenes was tested. The DCWs of the wild-type and its mutant, ppGpp^0^, were monitored in the GYM medium at 96 h at different concentrations (0 to 200 μg/mL) of gentamicin, kanamycin, streptomycin, and rifampin (Fig. 6). The results showed that the DCW of the ppGpp^0^ mutant was significantly higher than that of wild-type strain without the antibiotics (0 μg/mL), which was consistent with the previous results that the ppGpp^0^ mutant had faster cell division rate in GYM medium. However, at increased concentrations of antibiotics, the DCW of the wild-type strain was significantly higher than that of the ppGpp^0^ mutant, which indicated that the cell growth of the wild-type strain was faster than that in the ppGpp^0^ mutant. For the types of antibiotics at the same concentration (50 μg/mL), DCWs of *Streptomyces* in the gentamicin (0.81 g/L) and rifampin (0.88 g/L) were lower than the DCWs of strains in the kanamycin (1.88 g/L) and streptomycin (2.59 g/L), which suggested that the growth of S. diastatochromogenes was also affected on the types of antibiotics. When the strains were treated with a high concentration of antibiotics (200 μg/mL), the DCW of wild-type and ppGpp^0^ were below 0.5 g/L, indicating the inhibition effect of antibiotics. The results confirmed that the antibiotic resistance was related to the ppGpp-mediated stringent response in *Streptomyces*.

## DISCUSSION

The ppGpp-mediated stringent response is widely distributed in E. coli, Bacillus subtilis, *Streptomyces*, Staphylococcus aureus, etc. Stringent responses give strains the ability to resist environmental stresses, and survival from nutrition starvation, virulence, long-term persistence, biofilm formation, and gut colonization (21). During the stringent response, ppGpp concentration was controlled by the proteins of the Rel/SpoT homolog (RSH) family, and these enzymes could synthase and hydrolyze ppGpp (21). ppGpp has many targets in cells, which could reprogram DNA replication, transcription, ribosome biogenesis and function, and lipid metabolism (7). However, how ppGpp controls transcription and other metabolic processes depending on the bacterial species and regulates the same process via a different mechanism. For example, ppGpp inhibits the DNA replication initiation in E. coli by interacting with RNA polymerase while slowing down the elongation of DNA replication by targeting the primase (*DnaG*) in the Bacillus subtilis. In *Streptomyces*, how ppGpp controls the physiological characteristics remains to be elucidated. However, ppGpp certainly regulates many genes involved in primary and secondary metabolism (3). We, therefore, systematically compared the transcription and cell division, cell growth, morphological differentiation, antibiotic resistance, and secondary synthesis in the wild-type S. diastatochromogenes and ppGpp^0^ mutants. The deficiency of ppGpp could lead to higher cell division and growth rate in a nutrition-sufficient environment, which was similar to the growth of Pseudomonas protegens H78 and its Rel/SpoT mutant (22), but the ppGpp^0^ mutant led to a lower growth rate in the nutrition-limited condition. Therefore, the ppGpp-mediated stringent response used dedicated mechanisms to respond adequately to survival in harsh conditions.

In addition, morphological development during the growth and reproduction of *Streptomyces* has been proven to be associated with the ability to produce variable clinically relevant antibiotics. The *Streptomyces*’ morphological transitions from vegetative growth to the stationary phase are accompanied by the *Bld* genes, which also regulate the secondary metabolites synthesis (13, 14). Therefore, the *Streptomyces* mutants with high antibiotic production were often revealed with a change of morphological difference (23). In this study, we found the lack of ppGpp resulted in the change of *BldD* and *WhiA* transcription, indicating the ppGpp-mediated stringent response could regulate the developmental transitions.

In S. coelicolor, the ppGpp has been proven to be related to the increased transcription of activator gene controlling Act biosynthesis, actII-ORF4 (24), which means there is a link between ppGpp and secondary metabolic. However, in the same strain, the accumulation of ppGpp does not induce the undecylprodigiosin (RED) production, and ΔRelA or ΔRSH in S. coelicolor M600 leads to higher RED production (25). In addition, the production of clavulanic acid and cephamycin C was also increased in the *relA* mutant S. clavuligerus (11). Thus, there is uncertainty about the higher ppGpp levels and higher secondary metabolites. In this study, we tested the effect of ppGpp on the expression of the toy cluster and tetraene macrolide cluster and found that the ppGpp could positively regulate the production of toyocamycin and tetraene macrolides in the S. diastatochromogenes. Toyocamycin is a pyrrolopyrimidine nucleoside antibiotic, and the cluster to synthesize the toyocamycin contained 13 genes, starting from the GTP involved with NADPH (26). Although the synthesis of ppGpp could decrease the GTP pool, the increased transcription and yield of toyocamycin indicated there is another regulation mechanism to stable the GTP concentration *in vivo*. There are more than 200 kinds of tetraene macrolides in nature, and many of them were synthesized in *Streptomyces* (2, 27). In the S. diastatochromogenes, three types of tetraene macrolides were identified and the clusters were analyzed by BLAST and compared with the tetramycin P cluster in the S. hygrospinosus variant beijingensis (2). The transcription and yield of tetramycin P revealed that the ppGpp could control the production of the tetraene macrolides, which brings insight into improving the production of the tetraene macrolides in the *Streptomyces*.

Antibiotic tolerance and resistance were also linked to the synthesis of ppGpp. Upregulation of ppGpp occurred in the emergences of chronic resistance, for example, the stringent response induced the methicillin-resistance S. aureus strain from a chronically infected patient (28). In the RelA/SpoT mutant of P. protegens H78, the live cells were significantly inhibited compared to the wild-type strain in the antibiotic-tolerant condition (22), and the same phenomenon was also achieved in this study. Thus, when we consider the activation of antibiotic production, the ppGpp-mediated stringent response seems to be the linkage between antibiotic production and antibiotic resistance in the S. diastatochromogenes 1628. Although the mechanism of how ppGpp regulates the production and the resistance of antibiotics is still unclear, it is certain that screening antibiotic resistance mutants of *Streptomyces* could activate the silent clusters and generate valuable, novel, biologically active compounds (29). In addition, cumulative resistance mutations could improve antibiotic production dramatically in *Streptomyces* in several situations (10). Next, we needed to explain the detailed mechanism between the ppGpp and antibiotic production/resistance.

In conclusion, ppGpp-mediated stringent response affected RNA polymerase synthesis and ribosome assembling, growth, morphological differentiation, and nutrient uptake and improved the cell survival of S. diastatochromogenes 1628. Dedicated mechanisms in response to the environment were observed, and the ppGpp enabled the cell to survive harsh conditions (such as nutrition-limited and antibiotic conditions). The signal molecular ppGpp also activated the secondary metabolism and enhanced antibiotic resistance at the same time, suggesting that ppGpp is the link between antibiotic production and antibiotic resistance. This study made a systematic explanation of how ppGpp affects the *Streptomyces*, which will help to understand the function of ppGpp in the *Streptomyces*.

## MATERIALS AND METHODS

### Bacterial strains, plasmids, primers, and culture conditions.

S. diastatochromogenes 1628 was isolated and stored in the China General Microbiological Culture Collection Center (CGMCC no. 2060). E. coli JM109 was used to construct the plasmids. The pKC1139 was used to delete the *Rel* gene. The primers were listed in Table S1 in Supplemental File 1. All Escherichia coli strains were grown in the Luria Bertani (LB) and 2×yeast extract tryptone (2×YT) medium at 37°C supplemented with antibiotics if necessary. S. diastatochromogenes 1628 was cultured in the GYM medium and appropriate antibiotics were added at 28°C on a rotary shaker at 180 rpm. The methylation-deficient strain of E. coli, ET12567/pUZ8002, was used as the donor for plasmid transfer by intergeneric conjugation to *Streptomyces*. Conjugation and selection of exconjugants were performed on the solid mannitol soya flour (MS) agar medium for sporulation. The LA-Taq DNA polymerases, T4 ligase, DNA marker, and other related enzymes were purchased from TaKaRa, Kusatsu, Japan. The genome of S. diastatochromogenes, the plasmids extraction, and purification of DNA fragments was performed by relative kits according to the kit proposal from Kakara (Dalian, China).

### Construction of the ppGpp^0^ strain in S. diastatochromogenes 1628.

To construct in-frame, marker-free deletions of *Rel* (GenBank accession no. ON950080), the gene of interest flanked with two homology arms (~2 kb each) was amplified from the genomic DNA by PCR using the primer Rel-left-for/Rel-left-rev and Rel-right-for/Rel-right-rev (Table S1 in Supplemental File 1). The respective 3′ and 5′ ends of upstream and downstream fragments share 41 bp complementary regions. The homology arm fragments were mixed by the overlap PCR. Then, the fusion PCR product was inserted into the pKC1139 to generate pKC1139-arms. The pKC1139-arms were transferred to the wild-type S. diastatochromogenes 1628 by intergeneric conjugation. The cells were selected by the apramycin resistance (Apr^R^) first, then the selected cells were cultured at 37°C for homologous recombination. The Δ*Rel* was selected by apramycin sensitivity (Apr^s^). The mutants of Δ*Rel* were tested by the PCR method using the Rel-left-for/Rel-right-rev. The presence and stability of deletion of *Rel* in the *Streptomyces* were checked as described earlier (30). Clones were then selected, and *RshA* [HS] and *PbcSpc* [H] genes were sequenced (6). We found a frameshift mutant on the *RshA* gene. Thus, this clone did not have the ability to synthase ppGpp and was named ppGpp^0^.

### Transcriptional levels by RT-qPCR.

According to the growth curve of the S. diastatochromogenes 1628, the wild-type and ppGpp^0^ strains were cultured to the early, mid-, or late exponential phases in the GYM medium at 28°C. The samples were harvested by centrifugation at 4°C and 4000 × *g* for 10 min. The total RNA was extracted by the Takara MiniBEST Universal RNA Extraction Kit (Dalian, TaKaRa, Japan), and reverse transcribed using the PrimeScript RT reagent kit (TaKaRa, Kusatsu, Japan). qPCR was conducted in a 20 μL volume with 100 ng cDNA as the template using the SYBR Premix *Ex Taq* GC kit (TaKaRa, Japan), and the thermocycling conditions were as follows: 95°C for 5 min followed by 40 cycles of 95°C for 10 s, 60°C for 10 s, and 72°C for 30 s (primers are in Table S1 in Supplemental File 1). The 16S rRNA gene was used as the reference gene. Reverse transcription-quantitative (qRT)-PCR results were conducted in the Step One Plus Real-Time PCR system (Applied Biosystem), and the data were analyzed by the software Step One. The 16S rRNA gene processing protein (*rimM*) was used as a reference gene for normalization. The standard deviation (SD) indicates the standard deviation from three independent experiment replicates.

### Production of toyocamycin and tetraene macrolides.

The wild-type and ppGpp^0^ strains were cultured in the GYM medium with 2% inoculum size, and the samples were taken at intervals of 12 h. The production of toyocamycin and tetraene macrolides was analyzed by the high-performance liquid chromatograph (HPLC) method. The toyocamycin was performed on a Waters 2950 HPLC system, including a PDA detector. Then, 10 μL of the samples were injected into a column (RP-C18, 250 mm × 4.6 mm, 5 μm, Waters, USA) with a water-methanol gradient system with a flow rate of 1 mL/min. The methanol ranged linearly from 5% to 100% over 30 min and was then held for 10 min. The detection wavelength was 279 nm. The column temperature was maintained at 30°C.

The tetramycin P was determined using the RP-C18 (250 mm × 4.6 mm, 5 μm, Waters, USA) column, with the water-methanol gradient system, with a detection wavelength of 304 nm and a solvent flow rate of 1.0 mL/min. The gradient system ranged linearly from 15% to 100% methanol over 20 min and was then held for 8 min. The injection volume was 5 μL. The column temperature was maintained at 30°C.

### Assay for the resistance of antibiotics.

The gentamicin, rifampin, kanamycin, and streptomycin were selected to determine the influence of ppGpp on the antibiotic resistance of S. diastatochromogenes 1628. The wild-type and ppGpp^0^ mutant were cultured in the GYM medium containing the increased antibiotic concentrations (0, 50, 100, 150, and 200 μg/mL). After 96 h of shaking culture at 28°C, the DCW was measured.

### Scanning electron microscopy.

The wild-type and ppGpp^0^ mutants were cultured in the GYM medium at 28°C, and the hypha was collected and washed by the phosphate buffer (pH 7.4) twice. Then, the hypha was dissolved in the glutaraldehyde solution for 24 h. The samples were washed with the phosphate buffer (pH 7.0) twice, and an osmic acid solution of 1% was added for 1 to 2 h then washed with the phosphate buffer (pH 7.0) again. The gradient ethanol solutions (30%, 50%, 70%, 80%, 90%, 95%, and 100%) were used to dehydrate the samples. Then, the samples were treated with isoamyl acetate for 1 h. The samples were dried and coated at the tipping point. Finally, the sample was moved onto the cryostage in the main chamber of the microscope (SU8010, Hitachi), held at approximately −130°C, and viewed at 3 kV.

### Usage of different carbon and nitrogen resource.

To evaluate the influence of ppGpp on carbohydrate utilization and nitrogen utilization, the wild-type and ppGpp^0^ mutants were cultured in the inorganic basic medium supplied with different carbon and nitrogen sources. The inorganic basic medium contained 0.5 g/L NaCl, 0.5 g/L K_2_HPO_4_ × 3 H_2_O, 0.5 g/L MgSO_4_ × 7 H_2_O, 0.01 g/L FeSO_4_ × 7H_2_O, pH 7.5. The glucose, maltose, sucrose, lactose, and starch (20 g/L) were chosen as the sole carbon source in the inorganic basic medium. The NH_4_SO_4_, NH_4_Cl, and KNO_3_ (20 g/L), as the sole inorganic nitrogen, were added to the inorganic basic medium. To assess the ability of the wild-type and ppGpp^0^ mutants to utilize the organic nitrogen, the inorganic basic medium was supplemented with yeast extract, beef extract, and peptone (20 g/L). The DCWs were then measured after culturing for 4 days.

### Data availability.

The *Rel* gene sequence was available under NCBI (GenBank accession no. ON950080).
