# Supplementary material for: Physiology and Transcriptional Analysis of ppGpp-Related Regulatory Effects in Streptomyces diastatochromogenes 1628
Source: Microbiol Spectr. 2022 Dec 8;11(1):e01200-22. doi: 10.1128/spectrum.01200-22 (PMC9927088; doi:10.1128/spectrum.01200-22)
Supplement: Supplemental file 1 — Fig. S1 and Table S1. Download spectrum.01200-22-s0001.pdf, PDF file, 0.2 MB [file spectrum.01200-22-s0001.pdf]

**Physiology and transcriptional analysis of ppGpp-related regulatory effects in the *Streptomyces diastatochromogenes* 1628**

Yang Song, Xiangli Zhang, Zixuan Zhang, Xuping Shentu*, Xiaoping Yu*

Zhejiang Provincial Key Laboratory of Biometrology and Inspection and Quarantine, College of Life Science, China Jiliang University, Hangzhou 310018, China

***Correspondence:** Prof. Xuping Shentu; E-mail address: stxp@cjlu.edu.cn; Tel: +86-571-86876237

Prof. Xiaoping Yu; E-mail address: yxp@cjlu.edu.cn; Tel: +86-571-86836006

Table S1 Specific primers used in this study

| Primer | DNA sequence (5'-3') |
|---|---|
| Primers for ΔRSH mutant construction | |
| Rel-left-for | cgacggccagtgccaagcttatcaccaagcagacccgcttcac |
| Rel-left-rev | cgccgagcagcgggagaacctgacccgcaaggagccatggtatcgagcccatggc |
| Rel-right-for | tggccgcgcggcagccatggccatgggctcgataccatggctccttgcgggtcaggttctccc |
| Rel-right-rev | tatgacatgattacgaattctattggcgttgtgtagggtgcc |
| Primers for Real-time quantitative PCR | |
| ftsZ-for | Agtcatcaaggtcgtcggcat |
| ftsZ-rev | gtcggcgtcgctcatgagca |
| murF-for | Ggtggcctgttcgttgcgtt |
| murF-rev | gatcgccgggacgccg |
| wblA-for | Ccgcgtactcgtcgtcgaac |
| wblA-rev | cgacggtcacgtcatggcg |
| bldD-for | Tcagatctcgtcgtgctgcacg |
| bldD-rev | tcaggacgacctccgcacc |
| toyA-for | Catcgacctgcaagcggg |
| toyA-rev | ccggggtcggttccgag |
| toyC-for | Gcaacatgcaatcgagtggtcag |
| toyC-rev | cggtacgggacccgcc |
| toyG-for | Acgtggtccaggaaggtcag |
| toyG-rev | gggcatcgtcgagtacggc |
| adpA-for | Ggtaggccgcccggtag |
| adpA-rev | gctggagacctccgactattcg |
| tetC-for | Cggttctccggcggattcc |
| tetC-rev | aaggcttcccaggaggactcc |
| tetK-for | Tcgtcgaagttggggagactgaag |

| | |
|---|---|
| tetK-rev | aggaagtgctgcgggcc |
| tetR-for | Tataggcagcaccttggtggg |
| tetR-rev | gggcggatttgccgattcc |
| rpoB-for | Gcgaagatcaaggagcccctc |
| rpoB-rev | gtgggaacttcctgcccact |
| rpoC-for | Gagtgctactgcggcaagtacaag |
| rpoC-rev | gaagtaccagatgtgggtcacgg |
| rplK-for | Atgcctcccaagaagaagaaggtc |
| rplK-rev | ttgtaggccttgcagaactccatgat |
| rplY-for | Gatgtccttggcgagcacg |
| rplY-rev | ggagcacctgctcaacacg |
| rpsL-for | Gttccaggttagctttaccgagactg |
| rpsL-rev | aagtgcgggcgtcttgttcttc |
| rsmE-for | Cgtcttcctcgtcgaatcgct |
| rsmE-rev | cgccgtccgtcaggacga |
| rsmG-for | Acacccggctggtacgc |
| rsmG-rev | tggacacctccgtcgtgca |
| Ts-for | Gaccttgatgcgcgagaagc |
| Ts-rev | agggtcgcctcaacggc |
| pth-for | Gtgttgtacgtcgactgcgc |
| pth-rev | tgctgaaggacttctcgtccgc |
| fmt-for | Atgaggcttgtcttcgccg |
| fmt-rev | tcgggccgggtcacgac |

Figure

Figure Caption

**Figure S1** The intercellular ppGpp level in the wild-type (SD1628) and ppGpp0 mutant after inoculation for 24 h, 48 h, 72 h and 96 h. The cultured strains of 2 g (fresh weight) were frozen in liquid nitrogen and then grinded. Then 3 ml of 2 M formic acid were added and incubated for 30 min in ice-cold water. The following procedure, SPE extraction and ppGpp concentration measurement were same as the article (Ihara, Y, Journal of plant research, 2015).

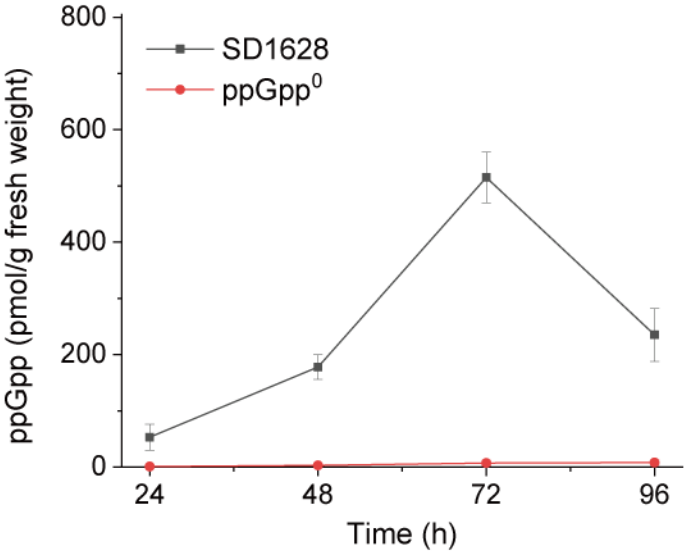

SD1628
ppGpp0
ppGpp (pmol/g fresh weight)
800
600
400
200
0
24
48
72
96
Time (h)
